# Stoking the Fire: How Dying Cells Propagate Inflammatory Signalling through Extracellular Vesicle Trafficking

**DOI:** 10.3390/ijms21197256

**Published:** 2020-10-01

**Authors:** Amy A. Baxter

**Affiliations:** Department of Biochemistry and Genetics, La Trobe Institute for Molecular Science, La Trobe University, Melbourne, VIC 3086, Australia; a.baxter@latrobe.edu.au

**Keywords:** extracellular vesicles, inflammation, cell death

## Abstract

Communication between dying cells and their environment is a critical process that promotes tissue homeostasis during normal cellular turnover, whilst during disease settings, it can contribute to inflammation through the release of intracellular factors. Extracellular vesicles (EVs) are a heterogeneous class of membrane-bound cell-derived structures that can engage in intercellular communication via the trafficking of bioactive molecules between cells and tissues. In addition to the well-described functions of EVs derived from living cells, the ability of dying cells to release EVs capable of mediating functions on target cells or tissues is also of significant interest. In particular, during inflammatory settings such as acute tissue injury, infection and autoimmunity, the EV-mediated transfer of proinflammatory cargo from dying cells is an important process that can elicit profound proinflammatory effects in recipient cells and tissues. Furthermore, the biogenesis of EVs via unique cell-death-associated pathways has also been recently described, highlighting an emerging niche in EV biology. This review outlines the mechanisms and functions of dying-cell-derived EVs and their ability to drive inflammation during various modes of cell death, whilst reflecting on the challenges and knowledge gaps in investigating this subgenre of extracellular vesicles research.

## 1. Introduction

Intercellular communication is a ubiquitous process that enables normal developmental, metabolic and immune functions. In addition to the many direct forms of communication between neighbouring or adjacent cells, including at the nerve synapse, the immunological synapse, during gaseous exchange and oocyte fertilisation, the phenomenon of extracellular vesicle (EV)-mediated cargo trafficking has come to represent a major source of communication between cells [1,2,3,4,5]. EVs are a heterogeneous class of membrane-bound cell-derived structures produced by all eukaryotic cells that can harbour bioactive molecules, including nucleic acids, lipids and proteins, derived from their parental cells [1,6]. Although once seen as inert cellular debris, a plethora of literature spanning numerous fields of medicine now describes EV-mediated cargo transfer that can directly modulate the functions of target cells and tissues [7,8,9,10]. Furthermore, EVs are increasingly recognised as potential diagnostic tools, serving as biomarkers for disease when isolated from biological fluids [11,12,13,14], whilst the therapeutic applications of stem-cell-derived EVs have demonstrated promising regenerative and immunomodulatory effects across a number of diseases [15,16,17].

Underpinning the body’s immune response to both acute and chronic pathological conditions is inflammation. Whilst a short-lived inflammatory response, such as during acute infection, acts to rapidly restore tissue homeostasis through immune cell recruitment and clearance mechanisms, chronic or aberrant inflammation, as seen during cancer, cardiovascular disease and neurodegeneration, can lead to tissue damage through prolonged immune signalling [18]. EVs derived from viable cells under conditions of stress or activation are well-described as mediators of inflammatory signalling [19]. For example, during atherosclerosis progression, the expression of cell surface adhesion markers on EC-derived EVs can promote vascular inflammation and immune cell migration [20], whilst during rheumatoid arthritis, platelet-derived microvesicles (MVs) harbouring proinflammatory cytokines can contribute to joint inflammation through the activation of synoviocytes [21] (reviewed further by Van Hazel and colleagues) [19]. However, what are the roles of EVs generated by dying cells in the propagation of inflammation? Direct communication between the released or exposed components of a dying cell and its surrounding tissue is a critical process that can promote efficient clearance of cellular debris and tissue repair during normal cell turnover [22,23,24] but can also contribute to inflammation during pathological processes such as infection and autoimmunity [25,26]. Importantly, in addition to these direct cell-to-cell interactions, emerging evidence now also highlights a role for EVs in the propagation of immune signalling in several cell death pathways. This review will discuss the features of dying-cell-derived EVs and how they can engage in proinflammatory intercellular communication during different forms of cell death. It should be noted that during inflammatory conditions, the transport of cargo by EVs derived from dying or inflamed cells can also mediate anti-inflammatory and regenerative effects on target cells, described elsewhere [27,28].

## 2. EV Diversity in Healthy and Dying Cells—Mechanisms of Biogenesis and Uptake

There are now four major classes of EVs described. Large oncosomes are the largest subfamily, ranging from 1–10 μm in diameter and shed from the plasma membrane of cancer cells [29]. As large oncosomes have not been observed during cell death, this review will be focused on the three other major EV categories: exosomes, microvesicles (MVs, also termed microparticles or ectosomes) and apoptotic bodies (ApoBDs). Although ApoBDs are the only EV subclass that, by definition, must be derived from dying cells, other types of EVs are also released during cell death via apoptosis, primary necrosis, secondary necrosis, pyroptosis and necroptosis, as summarised in Table 1 and discussed further below [30,31].

### 2.1. Biogenesis and Cargo Sorting

#### 2.1.1. Exosomes

Exosomes are 30–150 nm in size and are derived from the endosomal trafficking pathway, where late endosomes mature within multivesicular bodies (MVB) into interluminal vesicles (ILVs), which are then secreted by the cell as exosomes following MVB fusion with the plasma membrane [54]. Exosome cargo is sorted and packaged into ILVs via the endosomal sorting complex required for transport (ESCRT) machinery prior to MVB–plasma membrane fusion. There is also evidence for sorting to occur via an ESCRT-independent mechanism involving membrane tetraspanin and lipid-mediated processes [55]. Exosomes are enriched in proteins and lipids associated with their biogenesis, such as ESCRT-associated proteins Alix and TSG101, membrane tetraspanins, cholesterol, ceramide and sphingolipids. In addition, exosomes are well-known sources of nucleic acids, in particular miRNAs, and can also harbour other proteins and lipids derived from their parental cells [55]. 

#### 2.1.2. Microvesicles (MVs)

MVs range from 100–1000 nm and are formed through Ca^2+^-mediated changes in both the cytoskeletal protein and lipid composition of the plasma membrane, which promote the outward budding, pinching and release of the membrane to form distinct vesicles [55,56]. Ca^2+^-mediated scramblase activation has been shown to induce externalisation of phosphatidylserine (PS) during MV formation, whilst in cancer cells, where shed MVs have been extensively studied, the small GTPase RhoA and associated proteins, as well as GTP binding protein ADP-ribosylation factor 6 (ARF6), have been shown to contribute cytoskeletal rearrangements and membrane budding during MV formation [57,58]. MVs are enriched in ARF6 and small GTPase proteins as ARF6 has also been found to regulate MV cargo trafficking, where nucleic acids, nuclear proteins and membrane-associated proteins such as VAMP3 and b1 integrin may be sorted into nascent vesicles prior to release [56]. 

#### 2.1.3. ApoBDs

ApoBDs are typically 1–5 μm in size and are generated solely during apoptosis in a caspase-3/7-mediated step-wise fragmentation process known as “apoptotic cell disassembly”. Initial plasma membrane blebbing, regulated by rho-associated coiled-coil associated kinase 1 (ROCK1), is followed by the formation of plasma membrane protrusions, regulated positively by plexin B2 (PlexB2) and negatively by pannexin 1 (PANX1) [59,60,61]. Finally, the release of distinct ApoBDs from the cell occurs [60]. As with exosomes and MVs, ApoBDs are enriched in proteins associated with their biogenesis, including caspase 3-cleaved ROCK1, PlexB2 and PANX1 [61,62]. In addition, cytokines, growth factors and nuclear material have also been detected within ApoBDs [24,47,63]. 

#### 2.1.4. Alternative Nomenclature and Biogenesis during Cell Death

Due to significant overlap in size and cargo between EVs subfamilies, particularly exosomes and small MVs, accurate isolation of EVs from a mixed population into their distinct categories can be onerous. Therefore, simplified terminology such as “small EVs” and “large EVs” is also often used now to describe enriched populations, defined by size and/or density parameters set within the authors’ own methods of isolation [64,65]. It is also worth noting that during lytic forms of cell death, such as primary necrosis and pyroptosis where a specific EV biogenesis pathway is not determined, it is also possible that ruptured membrane fragments could reform into EVs extracellularly, as has been observed for dying Gram-negative bacteria [66].

In addition to exosomes, MVs and ApoBDs, there is now some evidence for alternative EV biogenesis pathways to be activated specifically during cell death [35,36,37]. For example, Park and colleagues observed a unique class of ESCRT-independent “exosome-like EVs” that formed during staurosporine-induced apoptosis and relied on a unique sphingosine 1-phosphate/sphingosine kinase 2 mechanism of biogenesis [35]. Interestingly, during necroptosis, induction of cell death processes has been explicitly linked to EV formation. The necroptotic membrane pore forming protein MLKL (mixed lineage kinase domain like pseudokinase; described in detail further on) has been implicated in the endosomal trafficking pathway, where its association with ESCRT machinery has been shown to facilitate ILV and EV formation. During necroptosis, MLKL has been found to promote the biogenesis of exosome-like necroptotic EVs [36]. Hence, it is now considered that EV release during necroptosis is a highly specific and regulated process. 

### 2.2. Uptake Mechanisms

**Figure 1:**
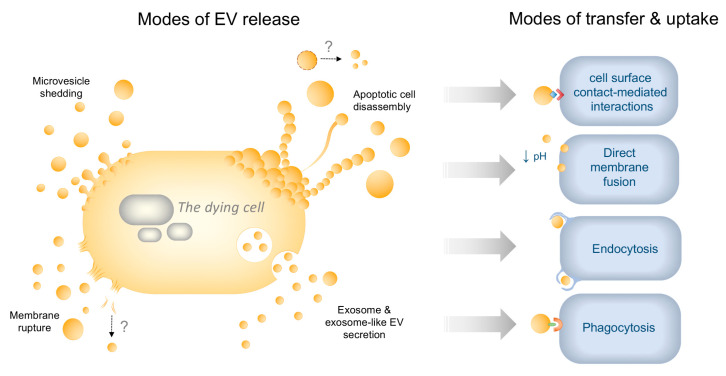
Modes of dying-cell-derived extracellular vesicle (EV) release and transfer to recipient cells. Dying cells can release EVs via a number of different mechanisms: (i) Microvesicle (MV) shedding involves the budding and release of MV (10–1000 nm) from the plasma membrane through Ca^2+^ mediated cytoskeletal changes; (ii) apoptotic cell disassembly induces the formation of apoptotic bodies (ApoBDs; 1–5 μm) released via caspase-dependent blebbing and protrusion formation during apoptosis. During progression to secondary necrosis, lysed ApoBDs could also reform into new EVs (denoted by ‘?’); (iii) exosome secretion occurs through the release of exosomes (30–100 nm) via the endosomal trafficking pathway. During both apoptosis and necroptosis, “exosome-like EVs” secreted via unique cell-death-associated mechanisms, have also been reported; (iv) during membrane rupture such as in primary necrosis, fragmented plasma membrane may also reform into EVs extracellularly (denoted by ‘?’). The release of undefined small, medium and large EVs (not shown) has also been described during various modes of cell death. EVs may then communicate with recipient cells through contact-mediated cell surface interactions, such as via expression of adhesion molecules on EVs facilitating binding to immune cells, or be taken up via direct fusion with the plasma membrane, which may be pH-dependent. Uptake can also occur via endocytosis or by phagocytosis, e.g., via expression of eat-me signals on EVs engaging with engulfment receptor phagocytes.

Once released, EVs can interact with other cells either by extracellular membrane surface interactions, direct membrane fusion, endocytosis or phagocytosis, although many of the specific events involved in the transfer of cargo from EVs to target cells remain to be elucidated [67]. EVs can influence the functions of cells within their environment even in the absence of uptake, such as through receptor-mediated adhesion events. Examples of this include the expression of tissue factor on EC- and leukocyte-derived MVs that can promote coagulation cascade events such as via a Factor VII binding and the expression of adhesion molecule ICAM-1 on EC-derived MVs, which can promote monocyte adhesion within the vasculature [68,69,70]. The uptake and transfer of small EVs into target cells have been proposed to occur via direct membrane fusion as well as various forms of clathrin-dependent and -independent endocytosis [67]. During direct membrane fusion, EV contents are released directly into the cytosol, which has been shown to be aided by acidic pH [71]. EV uptake via endocytosis events results in the formation of EV-containing endosomes that may fuse with EV membranes to release their contents intracellularly, target the EVs for lysosomal degradation or promote their rerelease via the plasma membrane [67]. Large EVs such as ApoBDs are generally considered to be phagocytosed by target cells (described further below), which occurs via interactions between eat-me signals such as externalised PS on the ApoBD membrane and engulfment receptors on phagocytosing cells, such as brain-specific angiogenesis inhibitor 1 (BAI1), growth arrest-specific 6 (Gas6) and milk fat globule EGF factor 8 (MFG-E8) on macrophages [22]. However, phagocytosis of smaller EVs has also been reported [72,73]. Phagocytosed EVs may then deliver cargo intracellularly or be directed to the phagolysosome for degradation. As with small endocytosed EVs, precisely how the delivery of functional phagocytosed EV cargo into target cells occurs relative to its degradation is largely unknown. Examples of the different modes of EV release and uptake are illustrated in Figure 1. It is worth noting that in addition to direct effects on target cells, EVs have also been described as mediators of extracellular matrix remodelling through the trafficking of matrix-degrading enzymes. This extracellular function of EVs has been implicated both in disease pathogenesis, including cardiovascular disease, cancer and arthritis, as well as during tissue regeneration (reviewed in detail by Nawaz and colleagues) [74].

## 3. Inflammatory EVs Generated during Different Modes of Cell Death

**Figure 2:**
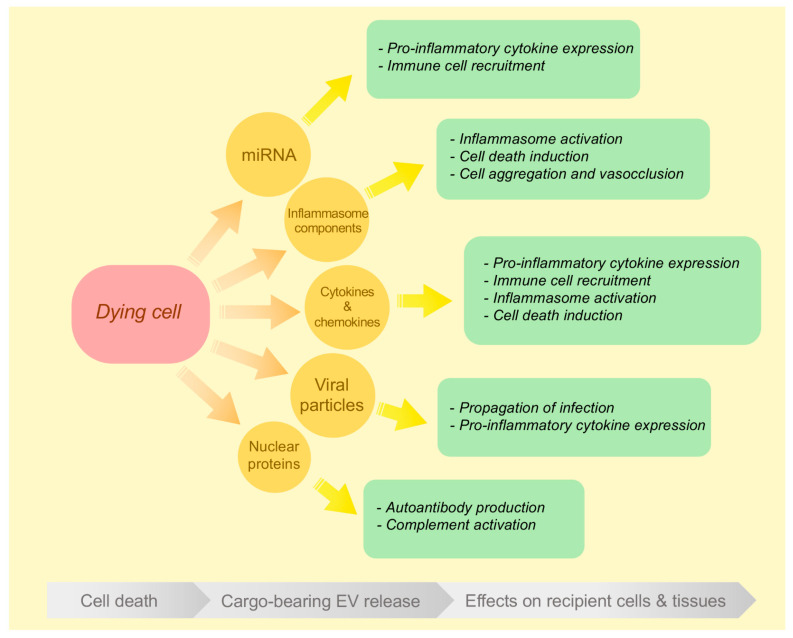
Dying cell-derived EV cargo and their proinflammatory effects on recipient cells and tissues. Schematic illustrating the various types of cargo trafficked by dying-cell-derived EVs and their respective modes of promoting inflammation through intercellular communication.

The following section describes evidence within the literature of the various proinflammatory effects mediated by dying cell-derived EVs during different modes of cell death and is summarised as a schematic in Figure 2. 

### 3.1. EVs Released during Apoptosis and Secondary Necrosis

Apoptosis is a highly regulated cell death pathway that occurs during both normal growth and development as well as during pathologies such as infection and autoimmunity [75]. Formation of the apoptosome by either extrinsic or intrinsic factors induces cleavage and activation of executioner caspases 3/7, leading to the processing of numerous cellular factors that, together, orchestrate cellular breakdown and promote the recruitment of phagocytes via find-me and eat-me signals [22,75]. Following successful engagement with phagocytic machinery on engulfing cells, efferocytosis can then take place, thereby preventing the release of intracellular damage-associated molecular patterns (DAMPs) such as ATP, nuclear proteins and heat shock proteins from the apoptotic cell and limiting the proinflammatory immune response [22]. In addition to these measures, phagocytosis of apoptotic cells by macrophages can also trigger the release of anti-inflammatory cytokines such as IL-10 and TGF-β, further dampening local inflammation [76]. In these ways, apoptosis is classically described as an immunologically silent process. Conversely, secondary necrosis occurs in the absence of successfully cleared apoptotic cells, when poor phagocyte mobility or sheer excess of apoptotic debris prevents sufficient phagocytic clearance prior to extensive cellular breakdown [77]. This can result in a proinflammatory response as intracellular contents such as DAMPs are released, which is observed during several pathologies including systemic lupus erythematosus (SLE), within the necrotic core of solid tumours, and atherosclerotic plaques [78,79,80]. As described above, cells undergoing apoptosis can disassemble into smaller fragments via a caspase-dependent series of steps that results in the formation of ApoBDs, a distinct class of EV. ApoBDs along with exosomes, MVs and exosome-like EVs are broadly known as ApoEVs. On the one hand, ApoEVs can act as regulators of homeostasis. ApoBDs and apoptotic MVs (ApoMVs) can aid engulfment through the display of find-me or eat-me signals derived from parental cells [72,81], whilst ApoBDs have demonstrated the ability to promote tissue repair and proliferation during development via the transfer of growth factors to neighbouring cells [24]. Furthermore, ApoMVs can promote vascular homeostasis through miRNA-mediated downregulation of adhesion molecules, while apoptotic exosomes can reduce intestinal inflammation through TGF-β signalling [82,83]. Intriguingly, a number of scenarios have also been reported in which ApoEVs can induce a proinflammatory response in target cells and contribute to deleterious effects on surrounding cells or tissues. 

The ability of ApoEVs to drive inflammation has been demonstrated predominantly via the transfer of cytokines to target cells, but the transfer of viral material, nuclear proteins and antibodies has also been reported. Exosome-like EVs derived from apoptotic HeLa cells following staurosporine treatment were shown to activate the NF-kB signalling pathway and induce Il-1β expression in THP-1 macrophages via uptake of sphingosine-1 phosphate 3 receptor expression on EV membranes [35]. In a model of nonalcoholic steatohepatitis, TRAIL-mediated apoptosis in hepatocytes caused by palmitate treatment promoted the generation of small EVs that could mediate a proinflammatory response in coincubated BMDMs. The ApoBDs were shown to contain TRAIL, which, when transferred to the macrophages, mediated IL-1β and IL-6 production through a RIP1-dependent, noncanonical TRAIL-mediated pathway [49]. EC-derived ApoBDs also promote inflammatory signalling in target cells. In a model of vascular inflammation, apoptotic HUVECs generated in the presence of TNFα released ApoBDs harbouring IL-1α, which promoted expression of IL-8 and MCP-1 in coincubated HUVECs and neutrophil recruitment in mice following intraperitoneal injection of the ApoBDs. [47] Importantly, it was suggested that IL-1α within the ApoBDs, which would normally be inactive and bound to chromatin in the parental apoptotic cell, was transferred to the HUVECs in its active form, suggesting that during their formation, ApoBDs may be loaded with a distinct set of cargo than that of their parental cells. ApoBDs can also propagate inflammatory signalling through the transfer of viral particles. For example, in an in-vivo model of Influenza A (IAV) infection, intranasal administration of THP1 monocyte-derived ApoBDs harbouring live IAV viral particles induced a robust proinflammatory cytokine and chemokine response in the lung tissue of mice [48]. 

A number of studies have also investigated the roles of EVs under conditions of dysregulated apoptotic cell clearance such as SLE, in which increased levels of circulating EVs are detected and can contribute to inflammation [40,41,42,84]. Elevated levels of circulating exosomes detected in SLE patient serum promoted TNFα, IL-1β and IL-6 production in coincubated peripheral blood mononuclear cells from healthy patients [40], whilst microparticles isolated from SLE patient serum contained elevated levels of IgG, associated with autoantibody and complement activation [42]. In another study investigating the role of EVs in autoantigen trafficking, HeLa cells were found to selectively package histone H2B into ApoMVs during cell death, further supporting a role for EV-mediated proinflammatory autoantibody production during SLE [43]. Furthermore, polymorphonuclear leukocyte-derived ApoEVs promoted IL-6, IL-8 and TNFα production in macrophages when coincubated in the presence of IFNa, a proinflammatory cytokine produced by dendritic cells that is detected in circulation at high levels in SLE patients [44]. 

Despite these studies describing EVs derived under conditions of poor clearance that are typically conducive to secondary necrosis, the question of whether cells committed to secondary necrosis can also generate EVs distinct from those released during the apoptotic phase has not been functionally addressed. However, quantitative characterisation of THP1 monocytes progressing from apoptosis to secondary necrosis over a 24-h period has been investigated, revealing a marked increase in small EVs, but not large EVs, in the postapoptotic phase of incubation [30]. Whether these EVs were generated through energy-dependent mechanisms, such as exosome formation or membrane shedding, or their specific cargo, was not determined. Furthermore, as it has been previously shown the ApoBDs can lyse over time [62]; the ability of ApoBDs undergoing lysis to subsequently reform into more EVs is also of interest.

Together, these studies indicate that while the expression of find-me and eat-me signals on apoptotic EVs can contribute to their phagocytic clearance, the specific environment in which a cell undergoes apoptosis, as well as the progression to secondary necrosis, may influence its cargo and, hence, its ability to trigger a proinflammatory response in target cells.

### 3.2. EVs Released during Primary Necrosis

Primary necrosis occurs in response to cellular injury, leading to irreversible plasma membrane damage such as by hypoxia, thermal injury or environmental toxins, and is defined by the absence of regulatory cell death processes [85]. The leakage of intracellular contents such as DAMPs through the ruptured or porous plasma membrane triggers a proinflammatory response in which the recruitment of immune cells migrate to the site of damage via target cell pattern recognition receptor (PRR) interactions. These processes then initiate engulfment and repair mechanisms and an eventual return to homeostasis [86]. The question of whether EVs released by primary necrotic cells can also contribute to inflammatory signalling, such as through the trafficking of cytokines, miRNA or DAMPs, has been investigated in a number of scenarios.

Acute injury of major organs such as heart, lung and brain are key examples of primary necrosis that can occur within the body. During acute myocardial infarction (AMI), levels of circulating exosomal miR-1 and miR-133a are detected in the hours immediately following injury. Experimentally, an increased concentration of miR-133a in exosomes corresponded with an increase in cell lysis, providing evidence that miR-133a is released specifically from dying cardiomyocytes [32]. In a murine model of AMI, both large and small EVs were found to be transiently released by cardiomyocytes over the 24 h following injury. Functionally, the EVs were engulfed by infiltrating LyC6+ monocytes, leading to increased monocytic expression of proinflammatory cytokine IL-6 and chemokines CCL-2 and CCL-7 [50]. Similarly, in a hyperoxia model of lung damage, injured EC-derived MVs harboured miR-221 and miR-320a, which led to elevated proinflammatory cytokine expression in vitro and the recruitment of alveolar macrophages in the lungs of mice exposed to hypoxia-induced MVs. In this study, the migration-enhancing effects of MVs were due to direct shuttling of the miRNA into recipient cells, which caused upregulation of the macrophage migration promoting enzyme matrix metalloproteinase-9 [45]. Likewise, patient sera collected within the first 24 h following stroke injury showed a significant increase in small EVs compared with age-matched controls. Proteomics analysis of isolated EVs revealed a more proinflammatory phenotype in the “stroke EVs”, while coincubation with THP-1 monocyte-derived macrophages induced elevated mRNA expression of proinflammatory cytokines TNFα, IL-1β and chemokines CXCL-1 and CCL-2 [51]. In addition to major organ injuries, EV release by cells undergoing primary necrosis following thermal stress has also been reported. An in-vitro kinetics analysis of EVs released by THP-1 human monocytes exposed to 56 °C heat stress demonstrated a time-dependent increase in EV release over a 45-min treatment period coinciding with an increase in LDH release, suggesting that EV generation occurred simultaneously to membrane lysis [30]. In this study, the heat-treated cells generated both large and medium EVs (isolated at 2000× *g* and 16,000× *g* centrifugation, respectively) and markedly fewer were isolated at 100,000× *g*, suggesting that the EVs were of nonexosomal origin [87]. In an in-vivo study on the role of EVs following major burns injury, analysis of blood samples of patients following thermal injury demonstrated elevated levels of circulating MVs that were predictive of mortality through their contribution to systemic inflammatory response syndrome (SIRS) [46], although the direct cause of this was not determined. Together, these findings support a role for EVs released during primary necrosis in propagating proinflammatory signalling, although the specific biogenesis of EVs generated under these conditions, as well as the identification of a primary necrosis-specific EV marker, requires further investigation.

### 3.3. EVs Released during Inflammasome Activation and Pyroptosis

Pyroptosis is an inflammatory cell death pathway activated in response to microbial infection as well as during sterile inflammatory pathologies [88,89]. A cell’s commitment to pyroptotic death culminates from initial cell surface receptor engagement with extracellular PAMPs, DAMPs or toxins, leading to PRR-mediated activation of one of several intracellular inflammasome complexes, the most well-studied being the NLRP3 inflammasome, which is comprised of nucleotide-binding domain leucine-rich repeat (NLR) and pyrin domain containing receptor 3 (NLRP3), apoptosis-associated speck-like protein containing a CARD (ASC) and pro-caspase 1. During inflammasome activation, cleavage of caspase 1 into its active form is responsible for both the activation of proinflammatory cytokines IL-1β and IL-18, as well as the N-terminal cleavage of gasdermin D, which then forms membrane pores leading to cell lysis [90]. The highly inflammatory nature of pyroptosis can rapidly lead to resolution of infection at the acute level, whilst inflammasome activation in chronic conditions such as HIV or obesity can result in a positive feedback loop of immune activation, resulting in prolonged inflammation and associated tissue damage [91,92]. 

During inflammasome activation, cytokine release has been reported to occur via both classic membrane secretion as well as gasdermin D pores, but there is now strong evidence that EVs are also a source of cytokine and other inflammasome component release [93]. EV-mediated transfer of active inflammasome components to target cells has been shown to occur in vitro and in vivo and typically induces both the production of proinflammatory cytokines and/or lytic cell death in target cells, indicating that EVs make a notable contribution to inflammasome-mediated immune signalling. For example, in J774 macrophages, exosome-mediated transfer of NLRP3, ASC and caspase-1 following LPS-mediated inflammasome activation induced LDH release in recipient endothelial cells [33], whilst exosomes containing IL-1β and NLRP3 from LPS/nigericin-mediated inflammasome-activated murine BMDMs also induced LDH release, as well as expression of proinflammatory cytokines, in coincubated BMDMs via activation of the NfkB signalling pathway [34]. Murine disease models have also demonstrated EV-mediated communication during inflammasome activation. A murine model of diabetes-associated nephropathy showed that D-ribose-mediated NLRP3 inflammasome activation in podocytes led to enhanced exosome-like EV generation and the release of EV-containing IL-1β via the modulation of lysosomal–sphingolipid pathway proteins, indicating a specific inflammasome-mediated mode of EV generation [37]. EVs derived from inflammasome-activated platelets containing IL-1β and caspase 1, present in the serum of LPS-treated mice in a sickle cell disease model, contributed to platelet–neutrophil aggregation and lung vasocclusion [52], providing an example of a direct pathological outcome in vivo that is mediated by inflammasome-derived EVs. In the sera of stroke patients, levels of serum-derived EVs harbouring IL-1β, IL-18, ASC and caspase 1 were significantly elevated [12], whilst the same group later reported that ASC-containing EVs from traumatic brain injury patients could propagate inflammatory signalling by inducing inflammasome activation and pyroptosis in lung endothelial cells [53]. It is important to note that the majority of the above examples did not directly report pyroptotic cell death occurring following inflammasome activation. Therefore, the possibility that EV generation preceded cell death, or that cell death did not occur, cannot be ruled out. However, in a study directly investigating the characteristics of pyroptotic EVs from THP-1 monocytes following nigericin/LPS treatment, the generation of EVs was monitored in parallel to membrane permeabilisation, demonstrating an abundance of small, medium and large EVs being released, coinciding with cell death [30]. These findings collectively indicate that interactions between cargo-bearing EVs derived from inflammasome-activated cells and immune cells contribute to an enhanced proinflammatory response.

### 3.4. EVs Released during Necroptosis

Like pyroptosis, necroptosis is a membrane-lytic and proinflammatory form of programmed cell death that occurs in response to microbial pathogens as well as during sterile inflammatory diseases such as atherosclerosis and nonalcoholic fatty liver disease [94,95,96]. Necroptosis can be activated by several extracellular ligands including TNFα, IFNγ and LPS, which, when caspase 8 is downregulated or rendered inactive either pharmacologically or through viral inhibition, can initiate the formation of a RIPK1–RIPK3 signalling complex. This complex then facilitates phosphorylation of MLKL, enabling its active translocation to the plasma membrane and the formation of membrane pores that can release DAMPs into the extracellular space [87,97]. There are relatively few examples of EVs released by necroptotic cells, although those that have been reported have provided intriguing insights into their unique properties [31,36]. The characterisation of EVs released by U937 cells undergoing necroptosis revealed that these cells externalised PS in an MLKL-dependent manner and released small EVs that also displayed PS prior to membrane permeabilisation and harboured MLKL from their parental cells [31]. It has been proposed that MLKL is shed into the PS^+^ EVs as a means of expelling MLKL-damaged membrane, delaying parental cell death and triggering recognition by the immune system [36,38]. The ability of necroptotic EVs to be phagocytosed was also confirmed by Yoon and colleagues, who demonstrated that the EVs could be efficiently engulfed by murine peritoneal macrophages, triggering a proinflammatory response through the secretion of IL-6, TNFα and CCL2 in the macrophages, following engulfment [31]. In a heat stroke model of hepatocyte injury, heat-stroked HepG2 cells released exosome-like EVs that were enriched in cell death and inflammatory signalling pathway proteins [39]. Coincubation of the EVs with hepatocytes induced expression of MLKL and RIPK3 in the target cells and caused both apoptosis and necroptosis. These observations were validated in vivo, where heat-stroked EVs injected into the liver of mice induced hepatocyte apoptosis and necroptosis [39]. Although the specific cargo driving each of these cell death pathways in recipient cells was not fully addressed, the study provided a striking example of the pleiotropic effects of EV-mediated communication from a particular cell type during cell death. Together, these studies highlight that necroptotic EVs are emerging as important mediators of proinflammatory signalling during cell death. 

## 4. Challenges in Reporting on Dying Cell-Derived EVs

As described within the findings of this review, while in vitro studies can readily demonstrate that an EV is derived from a dying cell via kinetics analyses, live-cell imaging or detection of death-associated markers, in vivo studies in which circulating EVs are isolated from biological fluids often rely on measuring changes in EV concentrations during specific disease states or following acute trauma [12,30,41]. Thus, a major obstacle in characterising dying-cell-derived EVs in in vivo studies is sufficiently isolating or enriching them from a mixed population [98,99]. Importantly, stimuli capable of inducing cell death may first promote a stress response preceding irreversible commitment to cell termination. Harmful stimuli, including thermal stress, inflammatory cytokine signalling and chemical toxins can cause the increased generation of extracellular vesicles in viable cells [20,100,101]. Therefore, in the absence of imaging or detection of death markers, determining whether an EV has been specifically generated during the process of cell death rather than during a stressed state preceding death or from a dying vs. viable cell of a mixed population requires rigorous interrogation, and it must be acknowledged that in the examples given herein, this was often not reported. Thus, a continued effort must be made within the field to ensure the integrity of reporting the source of EVs. 

## 5. Concluding Remarks

In recent decades, the pursuit of understanding the roles of communication between dying cells and their environment has contributed to our knowledge of many critical biological processes, including development, immunity and aging [78,102,103]. Importantly, dying-cell-derived EVs are now emerging as an important subgenre of cargo traffickers in the communication between cells. The generation of EVs occurs via the orchestrated rearrangement of endosomal-, cytoskeletal- and/or plasma-membrane-associated components. Intriguingly, the morphological changes that occur within dying cells can also contribute to their unique cell-death-specific biogenesis, cargo and effects on target cells [35,37]. As highlighted within this review, EVs derived from cells dying under inflammatory conditions are a source of functionally bioactive molecules that have demonstrated the ability to induce proinflammatory responses in target cells, predominantly via the transfer of cytokines, chemokines and miRNAs. Under conditions such as primary necrosis, the transfer of proinflammatory cargo from dying-cell-derived EVs to a target cell to promote inflammation may be viewed simply as an extension of the cell’s ability to expel its contents directly via a leaky or ruptured membrane. However, as detailed herein, there are now examples in which dying-cell-derived EVs possess distinct properties from their parental cells and can elicit unique immunomodulatory effects on target cells [47,52]. 

Whether a proinflammatory response within the body is advantageous or harmful is determined largely by how quickly inflammation is resolved. Cytokines and chemokines directly secreted from cells typically act on their local environment to recruit immune cells and promote cytokine expression [104]. However, as EVs are membrane-bound structures capable of travelling through the body, away from their parental cells, it is tempting to speculate that when released by cells during cell death and harbouring cytokines or other bioactive molecules, they may act over longer periods of time and traffic distally from their site of origin. How these tempospatial aspects of EV-mediated immune signalling are regulated, and how they affect the body’s overall immune response, remain to be determined. 

Finally, it is important to note that the release of EVs during the recently described cell death pathways of ferroptosis and NETosis was not addressed within this review due to the lack of current supporting evidence [105,106]. Since both ferroptosis and NETosis can induce plasma membrane lysis, it will be of interest in the future to determine whether EV release may also occur in these pathways and the specific roles such EVs could play in intercellular communication, including via proinflammatory signalling. 

In summary, the ability of EVs to propagate proinflammatory signalling is emerging as an important area of EV research that may provide valuable insights into the complexities of intracellular communication in years to come.

## Figures and Tables

**Table 1:** Types of EVs released by cells during different modes of cell death, as reported in literature.

EV Type	Apoptosis	Secondary Necrosis	Primary Necrosis	Pyroptosis	Necroptosis	Reference
Exosomes	-	-	+	+	+	[32,33,34]
Exosome-like EVs	+	-	+	-	+	[35,36,37,38,39]
Microvesicles	+	-	+	-	-	[40,41,42,43,44,45,46]
ApoBDs	+	-	-	-	-	[47,48]
Small EVs *	-	+	+	+	-	[12,30,31,49,50,51,52,53]
Medium EVs *	-	+	+	+	-	[30]
Large EVs *	-	-	+	+	-	[30]

‘+’ = ‘reported’, ‘-’ = ‘not reported’, ‘*’ = ‘EV type not determined’.
